# The Impact of Work Environment on Structural Empowerment among Nurses in Governmental Hospitals

**DOI:** 10.3390/nursrep14010037

**Published:** 2024-02-23

**Authors:** Abdalhady A. AL-Ghwary, Islam A. AL-Oweidat, Ahmad R. Al-Qudimat, Ghada M. Abu Shosha, Anas H. Khalifeh, Mohammed ALBashtawy

**Affiliations:** 1 Department of Community and Mental Health Nursing, Faculty of Nursing, Zarqa University, Zarqa 13110, Jordan; abdalghwary4@gmail.com (A.A.A.-G.); ioweidat@zu.edu.jo (I.A.A.-O.); ghada_abushosha@zu.edu.jo (G.M.A.S.); akhalifeh@zu.edu.jo (A.H.K.); 2 Surgical Research Section, Department of Surgery, Hamad Medical Corporation, Doha P.O. Box 3050, Qatar; 3 Department of Public Health, College of Health Sciences, QU-Health, Qatar University, Doha P.O. Box 2713, Qatar; 4 Department of Community and Mental Health Nursing, Princess Salma Faculty of Nursing, Al Al-Bayt University, Mafraq P.O. Box 25113, Jordan; mohammadbash@aabu.edu.jo

**Keywords:** work environment, structural, empowerment, nurses, hospitals

## Abstract

Background: The work environment is considered an important factor for the success of any healthcare organization that keeps upstanding and can compete with others to achieve the organization and employee’s goals. This study aims to examine the effect of the workplace environment on the structural empowerment of registered nurses in governmental hospitals. Methods: A cross-sectional, descriptive survey study. The sample consisted of 405 nurses from three Jordanian governmental hospitals. Data were collected using an online self-reported questionnaire that was distributed to the participants. The questionnaire included sections on demographic characteristics, the structural empowerment scale, and the work environment scale. Data collection took place between 1 January 2023 and 15 February 2023. Results: The participants demonstrated various levels in their perceptions of the working environment. They demonstrated a moderate perception level toward stress and work pressure, role clarity, peer cohesive subscale, and for working environment scale while they reported a low perception level on ethical, autonomy, work practices, managerial support, commitment, and social responsibility subscales. However, the nurses’ work environment overall score was found to be at a moderate level (3.15 out of 5 ± 0.61). Furthermore, their structural empowerment level was found to be at a moderate level (19.40 out of 30 ± 3.68). Conclusions: Highly empowered nurses’ work environments display higher structural empowerment. Applying structural empowerment in work environments is very important to improve nurses’ performance, ensure the highest level of patient care quality, and achieve organizational goals.

## 1. Introduction

Nowadays, the healthcare system is considered an uncertain area full of challenges and difficulties that require more attention and continuous development of plans and policies. These measures are necessary to cope with and survive in this dynamic environment and to keep pace with technological advances. The goal is to achieve high productivity and positive outcomes [1].

The work environment plays a crucial role in the success of any healthcare organization. It needs to be conducive and competitive to enable the organization to meet its goals and determine employees’ productivity and outcomes [1,2]. Moreover, it significantly impacts patients, affecting their safety, the quality of care they receive, and their overall outcomes [3]. However, the impact depends on the perception and type of work environment implemented, which affects patients, nurses, and the healthcare organization [2].

The work environment can be defined as the internal setting of an organization where employees carry out their work and interact with one another and with the organization [4]. For nurses, the work environment encompasses the organizational characteristics of the workplace that either enable or hinder professional nursing practice. This includes aspects such as the quality of care provided, nurses’ involvement in hospital policies, adequacy of staffing and resources, and the nature of nurse–physician relationships [5].

There are two types of work environments: collaborative and toxic. Collaborative work environments are characterized by friendliness, mutual support, empathy, and a sense of shared purpose with others [6]. On the other hand, toxic work environments are marked by negative behaviors, such as disrespect, poor leadership, threats, harassment, incivility, and bullying [7]. Toxic work environments not only contribute to physical and mental distress among employees, leading to negative psychological effects and increased stress levels, but also impact employee performance and productivity, ultimately leading to intentions to leave the organization [2,8]. The work environment has a significant impact on employees’ outcomes, whether positive or negative [1]. In the healthcare system, a positive work environment is crucial as it provides optimal working conditions for employees, leading to improved performance, job satisfaction, and innovation [9]. A safe and high-quality care environment contributes to better patient outcomes and reduced mortality rates [3,10]. Liu et al. (2021) have also emphasized the importance of supporting and enhancing the work environment to improve outcomes and promote quality care [11]. Creating a healthy work environment based on commitment, interpersonal relationships, and the application of work environment principles increases nurse satisfaction, performance, and the overall quality of care [12]. The work environment consists of three major sub-environments: the technical environment, the human environment, and the organizational environment. The technical environment encompasses tools, equipment, technological infrastructure, and other physical or technical elements that enable employees to perform their responsibilities and tasks effectively. The human environment includes interactions with peers, team members, and work groups, as well as leadership and management dynamics. It is designed to encourage informal interactions, knowledge sharing, and idea exchange, all of which contribute to maximizing productivity. The organizational environment encompasses systems, procedures, practices, values, and philosophies.

Management has control over the organizational environment, and issues within this environment directly impact employee productivity [13]. Within the organization’s work environment, nurses hold the responsibility for managing nursing care. This includes developing leadership practices, managing conflicts, staffing, and allocating resources to achieve high performance and positive outcomes. To support this, organizations should develop plans and programs to empower nurses, enabling them to become stronger and develop skills that promote positive changes in their work settings [14]. Empowerment is defined as giving authority, delegating authority, commissioning, or permitting individuals or groups to make choices and translate those choices into desired actions and outcomes. It involves building individual and collective assets, improving the efficiency and fairness of organizational and institutional contexts, and expanding the capabilities of individuals, particularly those who are marginalized, to participate in negotiating, influencing, controlling, and holding accountable institutions that affect their lives [15]. Empowerment is also about giving someone the authority or power to take action, making them stronger and more confident in controlling their lives and claiming their rights [16].

Empowerment enables individuals to approach experiences and challenges with a positive attitude. Empowered nurses are highly motivated and can inspire and empower others by sharing their sources of power. Empowering nurses not only creates a competitive workforce but also enhances client satisfaction and promotes a positive environment [17]. Therefore, this study aims to investigate the impact of the work environment on structural empowerment among nurses in Jordanian governmental hospitals.

## 2. Methods

A cross-sectional descriptive study was carried out at Jordan governmental hospitals between 1 January 2023 and 15 February 2023 year.

### 2.1. Design and Setting and Samples

The sample consisted of nurses working in all departments at 32 governmental hospitals from three different geographical regions (north, south, and central), selected in a random sampling method.

### 2.2. Sample Size

To determine the minimum sample size of the study participants, the sample size was estimated using the Thompson, KS formula: *n* = [*N* ∗ (1 − *p*)]/[*N* − 1 ∗ (*d*2/*Z*2)] + (1 − *p*), where *N* = population size (7525), *n* = sample size, *Z* = confidence level at 95%, *p* = probability (50%), and *d* = error proportion (0.05). Based on this formula, the minimum sample size is 366 nurses [18]. An additional 10% of participants were added to complete the incomplete questionnaire. Therefore, the sample size was 405.

### 2.3. Inclusion and Exclusion Criteria

The inclusion criteria were registered nurses in selected hospitals, those who have a Nursing Bachelor’s degree or more and have one year of experience at least. Therefore, they have adequate experience with the nursing profession. The exclusion criteria were other healthcare providers, and the nurse who refused to participate in the study.

### 2.4. Information Sources

An online self-report questionnaire was developed to measure structural empowerment. All questionnaires were administered in English without any changes or translation, as the nurses were familiar with the English language. The questionnaire consists of four parts.

The first part collects socio-demographic data, including general information about the participants such as age, sex, level of education, years of experience, region, and working area. The second part includes the structural empowerment scale condition for work effectiveness questionnaires II [19], and it contains 6 subscales: access to opportunity, access to resources, access to information, access to support, formal power, and informal power. A subscale mean score is obtained for each score by summing and averaging the items, and the range is between 1 and 5. Higher scores represent stronger access to the subscale. Total structural empowerment score is calculated by summing the six subscales; the response to each item may be 1 = none, 3 = some, and 5 = a lot. The range of total scores is located between 6–30; while 6–13 represent a low level of empowerment; 14–22 represent a moderate level of empowerment; and 23–30 represent a high level of empowerment. The third part represents the work environment using the Work Environment Services Scale (WESS), which was developed by Patrick & Kareem (2021) [20]. Approval was obtained from the author (4/2022). The scale consists of 9 dimensions and 32 items, including ethical, autonomy, stress and work pressure, work practices, managerial support, commitment, role clarity, social responsibility, and peer cohesiveness. Nurses respond using the following options: Strongly Disagree = 1, Disagree = 2, Neither Agree nor Disagree = 3, Agree = 4, Strongly Agree = 5. Cronbach’s coefficient was between 0.83–0.89 for the individual subscale, and 0.95 for the total scale. Moreover, the scoring interpretation is as following Table 1.

### 2.5. Statistical Analysis

Data were analyzed using SPSS software (IBM SPSS Statistics for Windows, Version 24.0; IBM Corp, Armonk, NY, USA). The analysis included descriptive statistics such as means, frequencies, standard deviations (SD), and percentages. The distribution of the data was assessed using the Single Sample Kolmogorov–Smirnov test and as the significance values exceeded 0.05, parametric tests were used in the advanced-level analysis. For parametric tests, *t*-tests were performed to analyze independent variables with two categories; Pearson’s correlation coefficients and multiple linear regression were used to analyze relationships.

### 2.6. Ethical Considerations

Prior to the commencement of data collection, approval was granted for the revised study proposal by both the Committee of Scientific Research and Ethics of Research at Zarqa University’s Faculty of Nursing and the Institutional Review Board (IRB) of the Ministry of Health. The study has been assigned an approval number (4/2022). All participants provided e-informed consent.

## 3. Results

Participant Characteristics

As shown in Table 1 the response rate of this study is 47%. Nurses employed in private hospitals were not included in the survey administration. Incomplete survey responses were excluded from the study. In total, 405 nurses responded. The mean age of the sample age was (34 ± 5.13) years and 41.2% were aged between 24–32 years. Moreover, 56.5% of the participants were female, 80.2% had a bachelor’s degree, 41.7% of them had experience from 11 to 15 years, and 43% worked in a critical unit. More than half of the sample (64.7%) of the participants were working in Al-Basheer Hospital (Table 2).

As shown in Table 3, nurses’ perception level toward their working environment ranges from low to moderate levels. However, the nurses’ perception toward ethics and ethical awareness, autonomy, work practices, managerial support, commitment, and social responsibility accounted for at a low level, while their perception toward stress and work pressure, role clarity, and peer cohesiveness scored at a moderate level. The nurses’ work environment overall score was found to be at a moderate level (3.15 out of 5 ± 0.61) (Table 3). Furthermore, the results have shown that the floor and ceiling effect was less than 15%, revealing no scores clustered to high or low ends. In other words, the nurses did not perform poorly or extremely on the work environment services scale. We found the correlation between structural empowerment and work environment; ethical, autonomy, managerial support, role clarity, social responsibility, and peer cohesiveness yielded a significantly positive weak correlation with structural empowerment. On the other hand, neither stress and work pressure, work practices nor commitment demonstrate any significant correlation with structural empowerment (Table 3).

As shown in Table 4, structural empowerment is composed of 6 sub-dimensions to describe the nurse’s ability to mobilize resources and achieve goals through accessibility. Similarly, descriptive statistics were used and the scoring system for categorizing the structural empowerment subscales levels relied on a 5-point Likert scale, while the interpretation of the total scale score was based on [18,21]. Structural empowerment subscales were at a moderate level. However, the access to opportunity subscale has the highest mean (3.38 out of 5 ± 0.98), and access to information has the lowest mean (3.08 out of 5 ± 1.05). In addition to the structural empowerment, the overall score was found at a moderate level as well (19.40 out of 30 ± 3.68) and no floor or ceiling effect has been identified in the structural empowerment scale (<15%). Furthermore, as a part of structural empowerment, the participants were presented with two items under the title global empowerment to rate how their work environment empowers them to accomplish the work and the results revealed the work environment empowers nurses at the moderate level (2.90 out of 5 ± 1.02) (Table 4).

In terms of the impact of each predictor on registered nurses’ structural empowerment scores, the findings revealed that nurses in the age group of 37 years or older and those with 1–5 years of working experience exhibited higher scores compared to nurses in the age group of 24–23 years and those with 11–15 years of experience (β = 1.362, *p* = 0.001) and (β = 1.133, *p* = 0.031), respectively.

As shown in Table 5, similarly, nurses working at Al-Karak Hospital reported higher structural empowerment scores compared to those working at Al-Basheer hospitals (β = 1.245, *p* = 0.010). Additionally, for every additional unit in the nurses’ work environment score, a corresponding increase of 0.875 units in structural empowerment is expected (β = 0.875, *p* = 0.003). Conversely, nurses working in the medical–surgical department exhibited lower structural empowerment scores compared to those working in the critical unit (β = −1.530, *p* < 0.001) (Table 5).

## 4. Discussion

This study’s findings revealed both similarities and differences when compared to the existing national and international literature as follows:

### 4.1. Perception Level of Work Environment among Jordanian Nurses in Governmental Hospitals

The results of the current study indicate that the overall score for nurses’ work environment was at a moderate level (3.15 out of 5 ± 0.61). This finding is consistent with Kretzschmer et al.’s (2017) study, which reported a moderate level of nurses’ work environment (2.08 out of 4 ± 2.2) [22] Similarly, a study that was conducted in Canada revealed an overall work environment score of 2.6 out of 4, indicating a moderately healthy work environment for nurses [23].

According to the current study’s results, nurses’ perceptions of their working environment ranged from low to moderate levels. This suggests a low level of effectiveness in handling grievances and a lack of involvement from managers, co-workers, and the work environment in ethical practices that impact and enhance employee behavior. Employees have limited freedom in planning and executing their work due to a work environment that restricts creativity, self-monitoring, and initiative. The management does not promote traditional work practices nor encourage new approaches and ideas. There is minimal encouragement from managers to support employees by considering their professional and personal needs and providing the necessary infrastructure for innovation and growth. Employees exhibit a lack of engagement with their organizations and jobs, expressing negative sentiments about them. The work environment does not exhibit socially responsible behavior, such as participating in charitable initiatives or providing opportunities for leisure and entertainment [24].

While the work environment indicates a moderate level in terms of the pressure created by demands such as meeting deadlines and managing expectations on employees’ work–life balance, there is also a moderate level of organizational clarity regarding employee expectations, responsibilities, and related processes. Additionally, there is a moderate level of interpersonal interaction within the work environment, including cohesion between workers, social communication exchange, and co-worker support [24] Previous research has shown that nurses prefer working conditions that offer greater opportunities for participation in decision making, increased autonomy, and strong relationships with physicians and other healthcare team members [22]. Nurses’ perceptions of control over their practice environment, autonomy, and good cooperation with physicians have also been found to be positively associated with a better workplace environment [21]. When nurses feel they have control in the workplace, their professional decisions are valued, and their work is seen as important and impactful, leading to increased job satisfaction and a sense of empowerment [25,26,27].

Supportive work environments have been consistently associated with nurses’ job satisfaction, high-quality nursing performance, and reduced rates of patient adverse events, including medication errors, nosocomial infections, and patient complaints [28,29]. Such environments enhance nurses’ perceptions of self-efficacy and competence, providing them with the confidence and abilities needed to meet job requirements and align with their mission’s core values [25]. Conversely, unfavorable work environments with increasing demands and limited job resources can lead to a decrease in nurses’ job morale, hindering their ability to exert greater effort in their work [30]. Due to resource constraints and heavy workloads, nurses may find it challenging to dedicate sufficient time to get to know patients and provide comprehensive care [19,29]. Effective leadership and managerial skills displayed by managers, along with the availability of resources and staff, contribute to nurses perceiving their work as autonomous and valuable [28]. Nursing managers play an active role in promoting feasible work environments and encouraging nurses’ participation in decision-making processes [25,30]. By assuming these roles, nurse administrators significantly influence nurses’ positive responses to their work and contribute to the enhancement of their autonomous practice.

Moreover, nurses who have access to adequate resources and staffing demonstrate increased confidence in their abilities, consider their jobs important, and experience a sense of control over their work [31]. When the work environment provides nurses with the necessary resources to fulfill their tasks, they feel that their efforts will lead to success and appropriate recognition, allowing them to find meaning in their work and work autonomously [32]. Sufficient resources and staffing also impact nurses’ ability to mobilize the necessary elements to complete tasks, thereby bolstering their confidence in their work [32]. Conversely, busy and understaffed work environments negatively affect nurses’ perception of patient engagement, as they may lack the time for therapeutic conversations and may be less inclined to activate patients [33,34].

The findings of the current study align with the previous literature, indicating the significant relationship between a favorable work environment and variables such as increased opportunities for decision-making participation, enhanced autonomy, control over the practice environment, cooperation with physicians, effective leadership, and managerial skills, and adequate resources and staffing. Conversely, the work environment exhibits a negative relationship with limited resources, increased demands, busyness, and heavy workloads. Therefore, these results reinforce the importance of the work environment in relation to nurses’ job satisfaction, high-quality nursing performance, and reduced rates of patient adverse events, as supported by previous research.

### 4.2. Level of Structural Empowerment among Jordanian Nurses in Governmental Hospitals

The results of the present study revealed that the overall score for structural empowerment was at a moderate level (19.40 out of 30 ± 3.68). These findings align with previous studies that also reported a moderate level of structural empowerment [33,34,35,36]. For example, Trus et al. (2018) found a moderate level of structural empowerment (21.0 ± 2.9) among nursing managers in Lithuania [36]. Similarly, Albasal et al. (2022) reported a moderate total structural empowerment score (20.5 ± 3.4) for Jordanian staff nurses [33].

In another study in Jordan, Darawad et al. (2020) assessed the perception of structural empowerment among newly qualified nurses and found a moderate level of perceived structural empowerment (11.92 ± 2.53) [34]. Saleh et al. (2022) conducted a study in Jordan analyzing nurses’ perception of structural empowerment and reported a moderate level overall score for structural empowerment (17.26 ± 6.15) [35]. Furthermore, Ta’an et al. (2020) conducted a Jordanian study that revealed a moderately perceived work environment in terms of structural empowerment (18.99 ± 5.06) among Jordanian nurses [37].

However, the results of the current study contradict the findings of previous studies that indicated a high level of structural empowerment. Aggarwal et al. (2018) conducted a study in India and found a high level of structural empowerment among Indian nurses, possibly due to organizations promoting a professional environment and supporting commitment [38]. The moderate level of structural empowerment observed in the current study suggests limited access to opportunities, support, resources, and information. It also indicates that Jordanian nurses’ internal sense of disempowerment influenced the level of structural empowerment revealed in this study.

Regarding the subscales of structural empowerment, the results of the current study indicated that the access to opportunity subscale had the highest score (3.38 out of 5 ± 0.98). This finding is consistent with the study by Trus et al. (2018), who reported the highest score for the access to opportunity subscale (4.0 ± 0.6) [36]. It suggests that participants in the current study perceived their work as providing opportunities to acquire new knowledge and skills. On the other hand, the access to information subscale had the lowest score (3.08 out of 5 ± 1.05). This indicates that nurses believed they had limited access to information related to the aims and values of their organization. Access to information plays a significant role in creating a positive work environment, promoting role satisfaction, and increasing feelings of empowerment, ultimately leading to the delivery of patient care and the achievement of organizational goals.

### 4.3. Predictive Factors for Structural Empowerment among Jordanian Nurses in Governmental Hospital

The results of the current study revealed a significant relationship between registered nurses’ age, years of working experience, working region, working department, work environment total score, and structural empowerment (*p* < 0.001). Nurses in the age group over 37 years old reported higher structural empowerment scores compared to nurses in the age group of 24–32 years old (*p* = 0.001). This finding is consistent with previous studies that also found a significant relationship between nurses’ age and structural empowerment, indicating that older nurses tend to have higher levels of structural empowerment compared to their younger counterparts [39,40]. This could be attributed to older nurses having more education and access to organizational resources, opportunities, and information, enabling them to align their tasks with organizational goals.

Furthermore, nurses with 1–5 years of working experience reported higher structural empowerment scores than nurses with 11–15 years of experience (*p* = 0.001). This finding contradicts previous studies that showed a positive relationship between working experience and structural empowerment, indicating that greater experience leads to higher structural empowerment scores [14,37,39,41]. The discrepancy in findings may be attributed to the composition of the current study’s sample, with nearly half of the participants falling within the 24–32 age range and 80.2% of them holding a bachelor’s degree, which may have influenced the structural empowerment scores. Additionally, Jordanian nurses with extensive experience might have limited access to updated knowledge due to a lack of workshops, continuous education opportunities, and specialized courses for improving and developing their information and skills. Moreover, nurses working at Al-Karak Hospital reported higher structural empowerment scores than nurses working at Al-Basheer hospitals (*p* = 0.010). This result can be attributed to Al-Karak Hospital not being a major referral hospital like Al-Basheer Hospital. The higher occupancy rate at Al-Basheer Hospital (95%) compared to Al-Karak Hospital (60.3%) [42], may contribute to the better conditions and resources available at the major hospitals, resulting in higher structural empowerment scores. Furthermore, the differentiation of nursing work in major hospitals, with specific situations and treatments provided, along with greater restrictions for sensitive patients, may contribute to the higher structural empowerment scores in those settings [43].

Similarly, nurses working in the medical–surgical department reported lower structural empowerment scores compared to nurses working in the critical care unit (*p* < 0.001). This finding aligns with previous studies that showed a significant relationship between nurses’ work setting and structural empowerment, indicating that nurses working in critical care units tend to have higher structural empowerment scores compared to those working in other units [14,23,41]. The dynamic nature of intensive care units, the complex and vulnerable patients they care for, and the high level of commitment and responsibility required from professionals contribute to the higher structural empowerment scores in these settings [14]. Additionally, positive perceptions of the work environment, collaborative decision-making, and a supportive atmosphere in the critical care units enhance nurses’ perception of their work environment [44].

## 5. Contribution of the Current Study

Nurses should support the creation of an empowered environment for hospitals by facilitating access to information and resources needed for nursing. Creating an empowered work environment encourages improved work environment, participation in decision making, increased internal opportunities, and improved quality of work life.

The current results might help nursing management in providing opportunities and support for nurses to grow, learn, and increase their influence in the workplace to motivate them and positively influence their job performance.

Furthermore, the current results might give clues to further researchers to tackle the issue of empowerment of nurses and the work environment qualitatively.

## 6. Limitations

Although this study has many strengths, many limitations were considered, including that the convenience sampling approach might limit the generalizability of the emerging results. In addition, this study relied on a self-reported questionnaire to collect data, which led to difficulties in verifying the responses of the participants and missing some data. In addition, it may lead to participants’ self-reported bias.

## 7. Conclusions

Registered nurses reported moderate levels of structural empowerment with a moderate level of the work environment. Highly empowered nurses’ work environments display higher structural empowerment. Healthy work environments play a key role in providing an outstanding quality of care, reducing staff turnover, and ensuring nurses’ job satisfaction. Applying structural empowerment in work environments is very important to improve nurses’ performance, ensure the highest level of patient care quality, and achieve organizational goals.

## Figures and Tables

**Table 1:** Scoring system for work environment service scale.

Dimensions	Very Low	Low	Moderate	High
Ethical Dimension	Below 2.75	2.75–3.5	3.5–4.00	Above 4
Autonomy	Below 2.8	2.8–3.4	3.4–3.8	Above 3.8
Stress and Work Pressure	Below 2.6	2.6–3.0	3.0–3.6	Above 3.6
Work Practices	Below 2.33	2.33–3.33	3.33–4.0	Above 4.0
Managerial Support	Below 2.75	2.75–3.5	3.5–4.0	Above 4.0
Commitment	Below 3.00	3.0–3.5	3.5–4.0	Above 4.0
Role Clarity	Below 2.33	2.33–3.0	3.0–3.75	Above 3.75
Social Responsibility	Below 2.66	2.66–3.33	3.33–4.0	Above 4.0
Peer Cohesiveness	Below 2.95	2.95–3.0	3.0–3.5	Above 3.5

**Table 2:** Characteristics of participants.

Variables	Label	N = 405
Age (mean ± SD)		34.0 ± 5.13
	24–32	167 (41.3%)
	33–36	137 (33.8%)
	More than 37	101 (24.9%)
Sex	Male	176 (43.5%)
	Female	229 (56.5%)
Level of Education	Bachelor’s degree	325 (80.2%)
	Graduate degrees	80 (19.8%)
Working Department	Medical–Surgical	116 (28.6%)
	Dialysis	17 (4.2%)
	Critical units	174 (43%)
	Others *	98 (24.2%)
Working Experience/Year	1–5	54 (13.3%)
	6–10	88 (21.7%)
	11–15	169 (41.7%)
	More than 15	94 (23.3%)
Working region	Al-Basheer Hospital	262 (64.7%)
	Prince Basma Hospital	81 (20%)
	Al-Karak Hospital	62 (15.3%)

* Other departments (operation ward, maternity ward, pediatric ward, recovery ward, clinics ward, administrators).

**Table 3:** Perception of work environment among Jordanian registered nurses in governmental hospitals.

Subscale	Min	Max	Mean	SD	Level	Floor %	Ceiling %	r-Coefficient	*p*-Value
Ethical (ethical practices)	1	5	3.34	0.88	Low	3.0	3.7	0.290	0.001 *
Autonomy (freedom in planning)	1	5	3.03	0.76	Low	2.0	1.2	0.219	0.001 *
Stress and work pressure (work–life balance)	1	5	3.35	0.76	Moderate	1.0	2.0	0.067	0.141
Work practices (encouraging new approaches and ideas)	1	5	2.93	0.84	Low	2.5	1.5	−0.052	0.169
Managerial support	1	5	3.09	0.76	Low	2.0	1.2	0.186	0.314
Commitment	1	5	3.02	0.93	Low	7.4	1.2	0.084	0.229
Role clarity	1	5	3.16	0.78	Moderate	2.5	1.0	0.161	0.002 *
Social responsibility	1	5	3.09	0.8	Low	2.5	2.0	0.108	0.944
Peer cohesiveness	1	5	3.17	0.91	Moderate	4.7	2.7	0.237	0.001 *
Total	1	5	3.15	0.61	Moderate	1.0	0.2	0.200	0.010 *

Min, Minimum; Max, Maximum; SD, Standard Deviation. * Significant.

**Table 4:** Level of structural empowerment among Jordanian nurses in governmental hospitals.

Subscale	Min	Max	Mean	SD	Level	Floor %	Ceiling %
Access to opportunity	2	5	3.38	0.98	Moderate	0.0	14.6
Access to information	1	5	3.08	1.05	Moderate	1.2	6.9
Access to support	2	5	3.32	0.81	Moderate	0.0	4.7
Access to resources	2	5	3.16	0.82	Moderate	0.0	4.7
Formal power	2	5	3.18	0.83	Moderate	0.0	6.4
Informal power	2	5	3.28	0.87	Moderate	0.0	6.9
Total	12	5	19.40	3.68	Moderate	0.0	0.0
* Global empotement (GE)	1	5	2.90	1.02	Moderate	7.7	3.0

* Not accounted for in structural scale scoring. Min = Minimum, Max = Maximum, SD = Standard Deviation.

**Table 5:** Impact of registered nurses’ socio-demographic data and work environment on structural empowerment score.

Final	Unstandardized Coefficients		Standardized Coefficients	t-Value	*p*-Value	Collinearity Statistics	
	β	SE	Beta			Tolerance	VIF
Age ≥ 37 vs. 24–32 years	1.36	0.415	0.160	3.280	0.001	0.923	1.084
Experience 1–5 vs. 11–15	1.133	0.522	0.105	2.170	0.031	0.945	1.058
Al-Karak vs. Al-Basher Hospital	1.256	0.483	0.123	2.602	0.010	0.985	1.015
Medical–surgical vs. critical units	1.530	0.0400	−0.188	−3.826	0.000	0.911	1.097
Work environment overall score	0.875	0.296	0.1444	2.954	0.003	0.922	1.084

F(6394) = 8.352, *p* < 0.001, Adj R2 = 9.8%, VIF = Variance Inflation Factors.

## Data Availability

All the data are available from the authors through reasonable request.

## Public Involvement Statement

No public involvement in any aspect of this research.

## Guidelines and Standards Statement

This manuscript was drafted against the Strengthening the Reporting of Observational Studies in Epidemiology (STROBE) guidelines for cross-sectional studies.
